# IL-33 Enhances IFNγ and TNFα Production by Human MAIT Cells: A New Pro-Th1 Effect of IL-33

**DOI:** 10.3390/ijms221910602

**Published:** 2021-09-30

**Authors:** Mourad Azzout, Céline Dietrich, François Machavoine, Pauline Gastineau, Alix Bottier, Guillaume Lezmi, Maria Leite-de-Moraes

**Affiliations:** 1 Laboratory of Immunoregulation and Immunopathology, INEM (Institut Necker-Enfants Malades), CNRS UMR8253, INSERM U1151, Université de Paris, 75015 Paris, France; azzoutM@outlook.fr (M.A.); celine.dietrich@inserm.fr (C.D.); francois.machavoine@parisdescartes.fr (F.M.); pauline.gastineau@inserm.fr (P.G.); alix.bottier@hotmail.fr (A.B.); guillaume.lezmi@aphp.fr (G.L.); 2 AP-HP, Hôpital Necker-Enfants Malades, Service de Pneumologie et Allergologie Pédiatriques, 75015 Paris, France

**Keywords:** IL-33, IL-12, inflammation, MAIT cells, innate-like T cells

## Abstract

Mucosal-associated invariant T (MAIT) cells represent a distinct T cell population restricted by the MHC-class-I-related molecule, MR1, which recognizes microbial-derived vitamin B2 (riboflavin) metabolites. Their abundance in humans, together with their ability to promptly produce distinct cytokines including interferon γ (IFNγ) and tumor necrosis factor α (TNFα), are consistent with regulatory functions in innate as well as adaptive immunity. Here, we tested whether the alarmin interleukin 33 (IL-33), which is secreted following inflammation or cell damage, could activate human MAIT cells. We found that MAIT cells stimulated with IL-33 produced high levels of IFNγ, TNFα and Granzyme B (GrzB). The action of IL-33 required IL-12 but was independent of T cell receptor (TCR) cross-linking. MAIT cells expressed the IL-33 receptor ST2 (suppression of tumorigenicity 2) and upregulated Tbet (T-box expressed in T cells) in response to IL-12 or IL-33. Electronically sorted MAIT cells also upregulated the expression of CCL3 (Chemokine C-C motif ligand 3), CD40L (CD40 Ligand), CSF-1 (Colony Stimulating Factor 1), LTA (Lymphotoxin-alpha) and IL-2RA (IL-2 receptor alpha chain) mRNAs in response to IL-33 plus IL-12. In conclusion, IL-33 combined with IL-12 can directly target MAIT cells to induce their activation and cytokine production. This novel mechanism of IL-33 activation provides insight into the mode of action by which human MAIT cells can promote inflammatory responses in a TCR-independent manner.

## 1. Introduction

Mucosal-associated invariant T (MAIT) cells are a special population of innate-like T (ILT) cells expressing an invariant TCRα chain, Vα19-Jα33 (TRAV1-2-TRAJ33) in mice and Vα7.2-Jα33 (TRAV1-2-TRAJ33) in humans [1,2,3,4]. They are restricted by the MHC-class-I-related molecule MR1 and recognize vitamin B2 (riboflavin) metabolites of microbial origin. MAIT cells play an important role in host defense against bacterial and viral infections by promptly producing IFNγ and TNFα [5,6,7,8]. MAIT cells secrete cytokines in response to TCR-dependent stimulation, but in the absence of riboflavin metabolites, they can also be activated directly by inflammatory cytokines, such as IL-12 and IL-18 [1,9]. 

We have previously demonstrated that invariant natural killer T (iNKT) cells, another ILT cell population, are targeted by IL-12 plus IL-18 [10]. This is also the case for MAIT cells [11]. Knowing that iNKT cells can respond to IL-12 plus IL-33 in a TCR-independent manner [12], we asked whether human MAIT cells could be similarly activated by IL-33. IL-33 is an alarmin implicated in inflammation, host defense, repair and homeostasis [13,14]. This alarmin is a tissue-derived nuclear cytokine from the IL-1 family abundantly expressed in endothelial cells, epithelial cells and fibroblast-like cells. IL-33 mediates its biological effects via interaction with the receptor ST2. This cytokine has emerged as a major regulator of tissue Tregs and ILC2s, but recent reports have highlighted its multiple roles in the regulation of immune cells and tissue responses [13,14]. Here, we report that IL-33 can activate and induce cytokine production by human MAIT cells.

## 2. Results

### 2.1. IL-33 Associated with IL-12 Activated Human MAIT Cells

**Figure 1:**
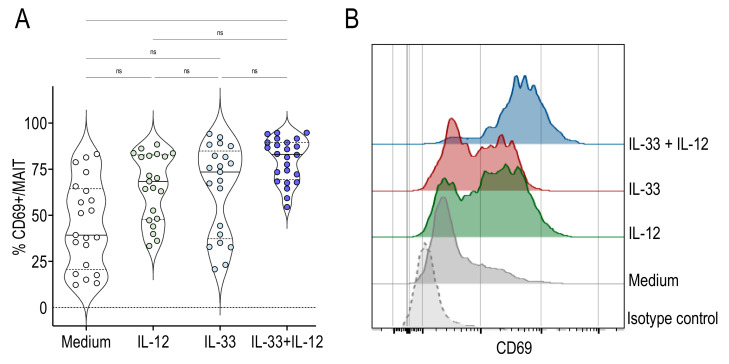
IL-33 + IL-12 activated human MAIT cells. (**A**) Percentages of CD69+ cells among gated MAIT cells stimulated for 48 h with IL-12, IL-33 or IL-33 + IL-12. Bars indicate medians and quartiles. Data were acquired from six donors in four experiments. (**B**) Histograms on the right are representative of CD69 expression among gated MAIT cells.

To determine whether IL-33 could activate human MAIT cells, we stimulated peripheral blood mononuclear cells (PBMC) from healthy adults with IL-33 alone or associated with IL-12. Activation of MAIT cells was assessed by the expression of the activation marker CD69. This marker is already present on the surface of MAIT cells and is further upregulated following stimulation. IL-12 or IL-33 alone tended to upregulate CD69 expression, whereas significant activation of MAIT cells was observed when both cytokines were present (Figure 1A). CD69 expression by stimulated MAIT cells from a representative donor is shown in Figure 1B.

### 2.2. Human MAIT Cells Promptly Produced IFNγ TNFα and GrzB in Response to IL-33 + IL-12 Stimulation

**Figure 2:**
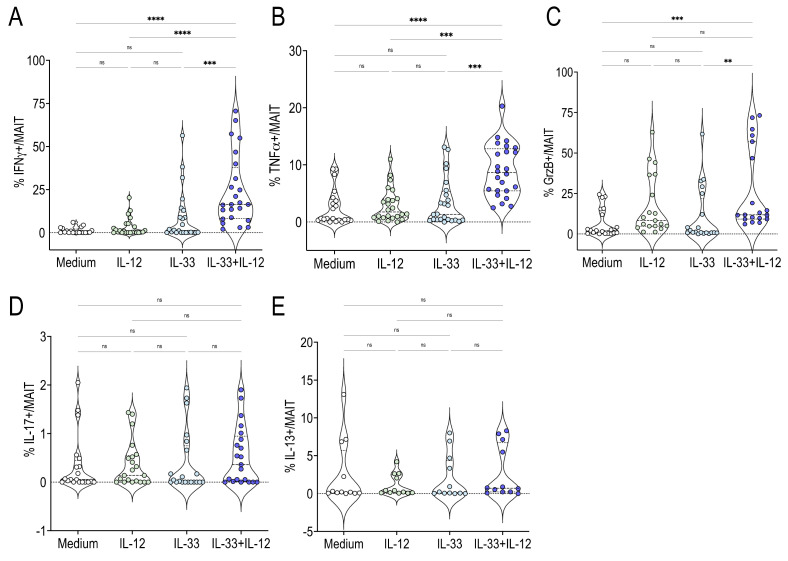
IL-33 + IL-12 induce IFNγ and TNFα production by human MAIT cells. (A,B,C,D,E) percentages of IFNγ + (**A**), TNFα + (**B**), GrzB+ (**C**), IL-17+ (**D**) and IL-13+ (**E**) cells among gated MAIT cells stimulated for 48 h with IL-12, IL-33 or IL-33 + IL-12. Bars indicate medians and quartiles. Data were acquired from six donors in two or four experiments. ** *p* < 0.01; *** *p* < 0.001; **** *p* < 0.0001.

Activated MAIT cells are able to promptly secrete cytokines, including IFNγ and TNFα. We found that human MAIT cells produced IFNγ and TNFα in response to IL-33 + IL-12, as assessed by intracellular staining (Figure 2A,B). IL-33 or IL-12 alone did not have this effect (Figure 2A,B). Previous studies have also reported that stimulated MAIT cells could secrete Granzyme B (GrzB), and, to a lesser extent, IL-17 and IL-13 [1,2,3,4,15]. MAIT cells stimulated with IL-33 + IL-12 also secreted GrzB (Figure 2C) but this was not the case for IL-17 or IL-13 (Figure 2D,E). 

**Figure 3:**
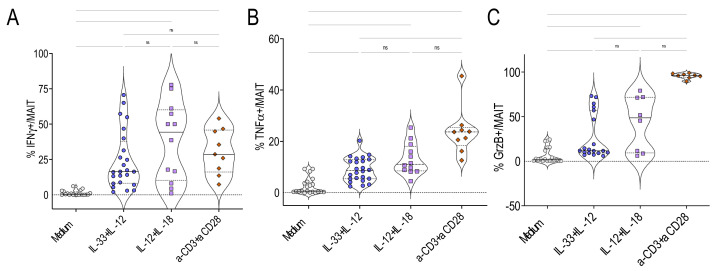
TCR, IL-33+IL-12 and IL-12+IL-18 promote cytokine production by human MAIT cells. (**A**–**C**) Percentages of IFNγ + (A), TNFα + (B) and GrzB+ (C) cells among gated MAIT cells stimulated for 48 h with IL-12+IL-33 or IL-12 + IL-18 or anti-CD3 + anti-CD28 beads. Bars indicate medians and quartiles. Data were acquired from four or six donors in two or four experiments.

The next step was to compare the stimulation by IL-33 + IL-12 with those obtained by IL-12 + IL-18 and by anti-CD3 + anti-CD28 beads as TCR triggers. The frequency of IFN*γ* + among gated MAIT cells stimulated with IL-33 + IL-12 or anti-CD3 + anti-CD28 beads was similar (Figure 3A). In contrast, the frequency of both TNFα + and Grz B+ among MAIT cells was significantly higher after TCR cross-linking compared with IL-33 + IL-12 stimulation (Figure 3B,C). No significant difference was observed between IL-12 + IL-18 and IL-33 + IL-12 or between IL-12 + IL-18 and anti-CD3 + anti-CD28 bead stimulations (Figure 3A–C). 

### 2.3. Human MAIT Cells Express the IL-33 Receptor ST2 in Response to IL-33 + IL-12 Stimulation

**Figure 4:**
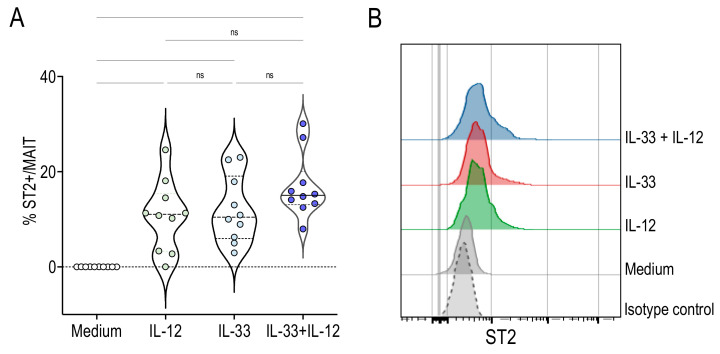
MAIT cells express ST2 following IL-33 or IL-12 stimulation. (**A**) Percentage of ST2^+^ cells among gated MAIT cells stimulated for 24 h with IL-12, IL-33 and IL-33 + IL-12. Bars indicate medians and quartiles. Data were acquired from five donors in two experiments. (**B**) Histograms on the right are representative of ST2 expression among gated MAIT cells.

Further, we examined whether MAIT cells expressed the IL-33 receptor ST2. The expression of ST2 was not detectable on MAIT cells cultured 48 h with medium (Figure 4). As shown in Figure 4*,* IL-12 or IL-33 alone promoted ST2 expression. The presence of both cytokines did not result in a significant increase compared with cytokines alone, but ST2 expression was strongly upregulated compared with the control medium (Figure 4). These results are consistent with previous reports showing that IL-33 could increase the expression of its own receptor by a positive feedback mechanism [16]. Furthermore, they demonstrate that both IL-12 and IL-33 induce ST2 expression by MAIT cells, making them responsive to this alarmin.

### 2.4. Activation of MAIT Cells by IL-33 + IL-12 Resulted in High Expression of Tbet

**Figure 5:**
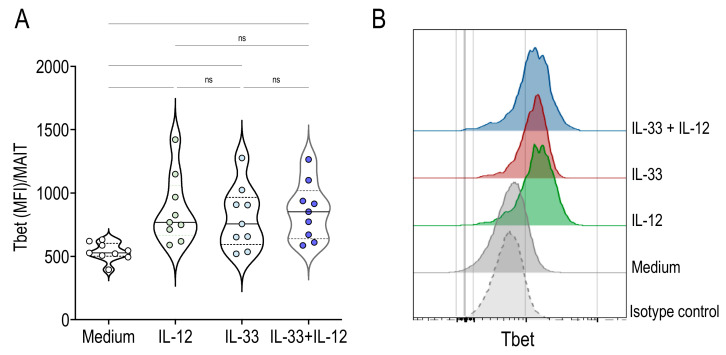
IL-33 and IL-12 enhanced Tbet expression by MAIT cells. (**A**,**B**) MFI of Tbet^+^ cells among gated MAIT cells stimulated for 24 h with IL-12, IL-33 or IL-33 + IL-12. Bars indicate medians and quartiles. Data were acquired from five donors in two experiments. Histograms on the right are representative of Tbet expression among gated MAIT cells.

Next, we examined the expression of the transcription factors promyelocytic zinc finger (PLZF) and Tbet by flow cytometry. PLZF is a specific transcriptional regulator of ILT cell development and function, while Tbet is critical for IFNγ production. MAIT cells expressed similar levels of PLZF in the presence of IL-33 and IL-12 alone or upon stimulation with both cytokines, compared with the medium control (2387 (2108-2913), 2583 (2156-3040, 2395 (2196-2880, 2468 (2105-2689), median and interquartile for PLZF (MFI)/MAIT following culture with IL-33, IL-12, IL-33 + IL-12 and medium, respectively). In contrast, Tbet expression by MAIT cells was significantly higher after stimulation with IL-12, IL-33 or IL-33 + IL-12 compared with medium (Figure 5). These results indicate that Tbet upregulation could be essential for IL-33-induced signal transduction in MAIT cells.

### 2.5. IL-33+ IL-12 Did Not Require Further TCR Cross-Linking to Activate MAIT Cells

**Figure 6:**
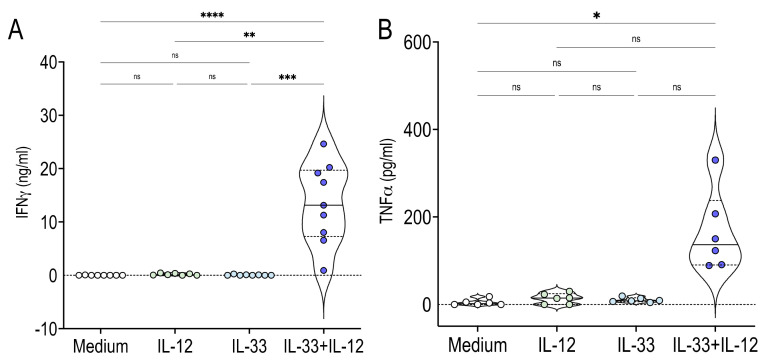
IL-33 + IL-12 induce IFNγ and TNFα production by sorted human MAIT cells. (**A**,**B**) IFNγ (**A**) and TNFα (**B**) production in supernatants from sorted MAIT cells stimulated for 48 h with IL-12, IL-33 or IL-33 + IL-12. Data were acquired from five donors in three experiments. * *p* < 0.05; ** *p* < 0.01; *** *p* < 0.001; **** *p* < 0.0001.

To assess whether IL-33 + IL-12 induced IFNγ and TNFα production by MAIT cells directly, without the contribution of other mononuclear cells present in PBMC, we electronically sorted MAIT cells and stimulated them with IL-12, IL-33 or IL-33 + IL-12. We found that IL-12 and IL-33 alone were unable to induce IFNγ (Figure 6A) and TNFα (Figure 6B) production. However, MAIT cells stimulated with IL-33 + IL-12 produced high levels of IFNγ (Figure 6A) and TNFα (Figure 6B). Together, these results clearly demonstrated that no further stimulation was required for IL-33 + IL-12 to stimulate MAIT cells. 

### 2.6. MAIT Cells Activated by IL-33+ IL-12 Expressed a Pro-Th1 Profile

**Figure 7:**
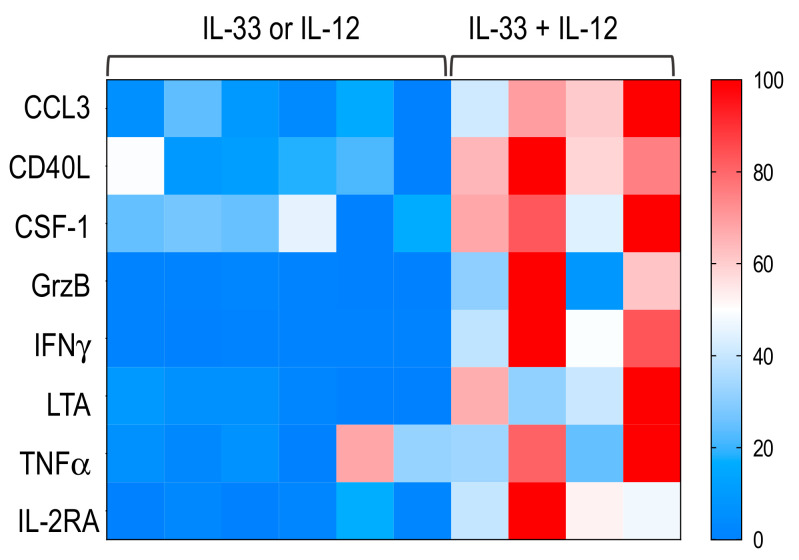
Sorted MAIT cells upregulated several pro-inflammatory factors following IL-33 + IL-12 stimulation. Heatmap showing a selection of significant mRNA expression enriched in sorted MAIT cells stimulated for 18 h with IL-33 + IL-12 compared to IL-33 or IL-12. Details are presented at Table S1.

We then examined the immune profile of sorted MAIT cells stimulated with IL-33 + IL-12. In addition to the expected increase in IFNγ and TNFγ, we also found that, among the 44 detectable genes analyzed, IL-33 + IL-12 induced a significant increase in the expression of mRNAs encoding CCL3, CD40L, colony stimulating factor 1 (CSF-1), GrzB, lymphotoxin A (LTA) and IL-2RA compared with IL-13 or IL-12 (Figure 7 and Table S1).

## 3. Discussion

MAIT cells can be stimulated in a TCR-dependent and TCR-independent manner [1,2,3,4,9,17,18]. IL-12 associated with IL-18 has been described as activating MAIT cells independently of the TCR [9]. A previous study reported that murine MAIT cells from Vα19iTg mice could secrete IFNγ in response to both IL-12 + IL-18 and IL-12 + IL-33 stimulations [19]. Here we showed that IL-33 combined with IL-12 activated human MAIT cells. The cytokine concentrations used to stimulate MAIT cells could not correspond to concentrations produced in situ during inflammatory responses. In addition, we cannot exclude the possibility that other inflammatory cytokines could also activate MAIT cells when associated with IL-33. The frequency of IFNγ-, TNFα- and GrzB-producing MAIT cells induced by IL-33 + IL-12 was similar to that obtained following IL-12 + IL-18 stimulation. IL-33 and IL-18 are members of the IL-1 family and are potent modulators of inflammation [13,14,20,21,22]. Knowing that IL-18 can be produced by inflamed macrophages, whereas IL-33 will be released mainly from damaged endothelial cells, epithelial cells or fibroblasts, we can postulate that these cytokines may have complementary functional consequences in the activation of MAIT cells. 

IL-33 is generally considered as a pro-Th2 cytokine [22,23]. However, we found no significant effect of IL-33 + IL-12 on IL-13 production by MAIT cells. The combination IL-33 + IL-12 did not induce detectable IL-17A secretion by MAIT cells. Previous studies have shown that tissue MAIT cells can secrete higher levels of IL-13 or IL-17 than their circulating counterpart [1,24,25]. Therefore, it cannot be excluded that IL-33, may also act on specific tissue MAIT cells to promote the production of pro-Th2 or pro-Th17 cytokines. Further studies are needed to assess this hypothesis. 

Previous studies indicated that the role of IL-33 in vivo is more complex than expected. In fact, IL-33 can suppress murine colon cancer growth and metastasis by upregulating CD40L and promoting IFNγ production [26]. Here we reported that IL-33 could increase Tbet expression by MAIT cells. Tbet is an essential transcription factor for optimal IFN**γ** production [27]. However, IL-33 alone was not able to induce IFN**γ** production, suggesting that IL-12 provides others signals to enable IFN**γ** secretion by MAIT cells. Further studies are needed to better understand the mechanisms involved.

A target analysis of a set of immune factors potentially produced by MAIT cells revealed that IL-33 + IL-12 also significantly increased the mRNA expression of CD40L, CCL3, CSF-1, LTA and IL-2RA. The increased expression of these effector genes by electronically sorted MAIT cells clearly shows that their response to IL-33 + IL-12 stimulation goes beyond the production of IFN**γ** and TNFα. Taken together, these data confirm the activation of MAIT cells by IL-33 + IL-12 and further indicate that IL-33, through its action on MAIT cells, could promote pro-Th1 inflammatory immune responses. 

MAIT cells play an important role in antibacterial responses, potentially through their ability to recognize bacterial metabolites presented by MR1 molecules [6,28,29]. They can also contribute to antiviral responses [30,31,32,33]. Although viruses cannot stimulate MAIT cells in a TCR-dependent manner, it is likely that cytokines present in the infected tissues could contribute to the activation of MAIT cells. This observation is interesting in the context of the current SARS-CoV-2 epidemic, which has revealed the importance of the “cytokine storm” in severe forms of infection [34]. These gravely affected patients are likely to produce alarmins, such as IL-33, as well as IL-12 [35,36]. It has been reported that the number of MAIT cells, which account for up to 10% of circulating CD3+ T cells, decreased significantly in peripheral blood from COVID-19 patients, while their activation was associated with poor clinical outcome [37]. It is also noteworthy that IFN**γ**- and TNFα-producing MAIT cells were more numerous in COVID-19 patients who died compared with those who recovered [37]. These observations and our results suggest that cytokines such as IL-33 and IL-12 may play a role in MAIT activation in severe COVID-19 patients. Future studies are needed to better understand the possible interactions between these cytokines and MAIT cells in these patients.

In conclusion, our results demonstrate that IL-33 + IL-12 induce IFN**γ**, TNFα and Grz production by human MAIT cells. This novel TCR-independent stimulation will open new avenues to explore for a better understanding of the immunopathological role of MAIT cells.

## 4. Materials and Methods

### 4.1. Subjects

PBMC from healthy donors were obtained from the French blood agency (Etablissement Français du Sang, EFS, Paris, France). Blood samples were collected with specific written informed consent from the donor for research use. PBMC were isolated using Ficoll-Paque density centrifugation (1.077 g/mL; PAA Laboratories GmbH, Cölbe, Germany). Cells were cryopreserved using DMSO/FCS solution and conserved at −150 °C until use. 

### 4.2. In Vitro Culture

PBMC and sorted MAIT cells were cultured at a density of 4 × 10^6^ and 4 × 10^5^ cells/mL, respectively, in RPMI 1640 medium supplemented with antibiotics, 10% FBS and 200 mM glutamine (GIBCO, Fisher Scientific SAS, Illkirch, France). Cells were stimulated for 48 h with IL-33 (50 ng/mL), IL-12 (50 ng/mL), IL-33 plus IL-12 (R&D Systems, Bio-Techne, Rennes, France) or anti-CD3 + anti-CD28 beads (Miltenyi Biotec Inc., Paris, France), and brefeldin A (10 µg/mL; Sigma-Aldrich, Darmstadt, Germany) was added for the last 4 h. In some experiments, cells were collected 18 h or 24 h after stimulation.

### 4.3. Flow Cytometry

Cells were stained with fixable viability dye (eBioscience-Thermo Fisher Scientific, Bleiswijk, The Netherlands) and then with the following antibodies for surface staining: anti-TCRVα7.2, anti-CD161, anti-CD3, anti-ST2, anti-CD4 and anti-CD8 (SONY biotechnology, Weybridge, United Kingdom) (eBiosciences-Thermo Fisher Scientific) (Biolegend, Amsterdam, The Netherlands). MAIT cells were identified as TCRVα7.2^+^CD161^+^ or MR1 5-OP-RU tetramer^+^ cells. MR1 6-FP tetramer^+^ was used as negative control (NIH Tetramer Facility, Atlanta, GA, USA) (Figure S1). For intracellular cytokine staining, cells were fixed in 4% paraformaldehyde, washed, and permeabilized with 0.5% saponin (Sigma-Aldrich), and then incubated with anti-IFNγ, anti-TNFα, anti-Granzyme B or isotype controls (eBiosciences-Thermo Fisher Scientific, SONY biotechnology or BioLegend). In some experiments, MAIT cells were stained intracellularly with anti-Tbet (BD Biosciences, Grenoble, France) and anti-PLZF (R&D Systems) antibodies using a transcription factor buffer set (BD Biosciences). Events were acquired on a FACS LSR Fortessa (BD Biosciences) and analyzed using FlowJo software v10.7.1 (Becton, Dickinson and Company, Ashland, OR, USA).

### 4.4. Cell Sorting and Cytokine Measure

CD3^+^CD161^+^TCRVα7.2^+^ MAIT cells were electronically sorted using a FACSAria (BD Biosciences). Sorted MAIT cells were then stimulated with IL-33, IL-12, IL-33 + IL-12 or medium. Two days later, supernatants were obtained. Cytokines were measured using DuoSet ELISA (R&D Systems) according to the manufacturer’s instructions. 

### 4.5. Real-Time Quantitative PCR

Total RNA was extracted 18 h after in vitro stimulation using the RNeasy Micro kit (Qiagen, Courtaboeuf, France). The cDNA was then synthesized using a high-capacity RNA-to-cDNA kit (Applied Biosystems). Real-time quantitative PCR was performed using a TaqManTM array human immune panel according to the manufacturer’s protocol (Thermo Fisher Scientific). Only detectable genes were represented at Table S1. Reactions were performed in triplicate and data were normalized to internal standards (ACTB, GAPDH, GUSB and PGK1) and expressed as relative expression using the **∆∆**Ct method versus the reference sample.

### 4.6. Statistical Analysis

Differences between data sets were analyzed by Kruskal–Wallis test, except for real-time quantitative PCR data, which were analyzed by Mann–Whitney using GraphPad Software, LLC (Prism 9.2).

## Data Availability

The data used to support the findings of this study are available from the corresponding author upon request.

## Supplementary Materials

The following are available online at https://www.mdpi.com/article/10.3390/ijms221910602/s1.

## References

[1] Godfrey D.I., Koay H.F., McCluskey J., Gherardin N.A. (2019). The biology and functional importance of MAIT cells. Nat. Immunol..

[2] Legoux F., Salou M., Lantz O. (2020). MAIT Cell Development and Functions: The Microbial Connection. Immunity.

[3] Victor J.R., Lezmi G., Leite-de-Moraes M. (2020). New Insights into Asthma Inflammation: Focus on iNKT, MAIT, and gammadeltaT Cells. Clin. Rev. Allergy Immunol..

[4] Toubal A., Nel I., Lotersztajn S., Lehuen A. (2019). Mucosal-associated invariant T cells and disease. Nat. Rev. Immunol..

[5] Treiner E., Duban L., Bahram S., Radosavljevic M., Wanner V., Tilloy F., Affaticati P., Gilfillan S., Lantz O. (2003). Selection of evolutionarily conserved mucosal-associated invariant T cells by MR1. Nature.

[6] Kjer-Nielsen L., Patel O., Corbett A.J., Le Nours J., Meehan B., Liu L., Bhati M., Chen Z., Kostenko L., Reantragoon R. (2012). MR1 presents microbial vitamin B metabolites to MAIT cells. Nature.

[7] Le Bourhis L., Dusseaux M., Bohineust A., Bessoles S., Martin E., Premel V., Core M., Sleurs D., Serriari N.E., Treiner E. (2013). MAIT cells detect and efficiently lyse bacterially-infected epithelial cells. PLoS Pathog..

[8] Wong E.B., Ndung’u T., Kasprowicz V.O. (2017). The role of mucosal-associated invariant T cells in infectious diseases. Immunology.

[9] Ussher J.E., Bilton M., Attwod E., Shadwell J., Richardson R., de Lara C., Mettke E., Kurioka A., Hansen T.H., Klenerman P. (2014). CD161++ CD8+ T cells, including the MAIT cell subset, are specifically activated by IL-12+IL-18 in a TCR-independent manner. Eur. J. Immunol..

[10] Leite-De-Moraes M.C., Hameg A., Arnould A., Machavoine F., Koezuka Y., Schneider E., Herbelin A., Dy M. (1999). A distinct IL-18-induced pathway to fully activate NK T lymphocytes independently from TCR engagement. J. Immunol..

[11] Brigl M., Bry L., Kent S.C., Gumperz J.E., Brenner M.B. (2003). Mechanism of CD1d-restricted natural killer T cell activation during microbial infection. Nat. Immunol..

[12] Bourgeois E., Van L.P., Samson M., Diem S., Barra A., Roga S., Gombert J.M., Schneider E., Dy M., Gourdy P. (2009). The pro-Th2 cytokine IL-33 directly interacts with invariant NKT and NK cells to induce IFN-gamma production. Eur. J. Immunol..

[13] Liew F.Y., Girard J.P., Turnquist H.R. (2016). Interleukin-33 in health and disease. Nat. Rev. Immunol..

[14] Cayrol C., Girard J.P. (2018). Interleukin-33 (IL-33): A nuclear cytokine from the IL-1 family. Immunol. Rev..

[15] Kelly J., Minoda Y., Meredith T., Cameron G., Philipp M.S., Pellicci D.G., Corbett A.J., Kurts C., Gray D.H., Godfrey D.I. (2019). Chronically stimulated human MAIT cells are unexpectedly potent IL-13 producers. Immunol. Cell Biol..

[16] Guo L., Wei G., Zhu J., Liao W., Leonard W.J., Zhao K., Paul W. (2009). IL-1 family members and STAT activators induce cytokine production by Th2, Th17, and Th1 cells. Proc. Natl. Acad. Sci. USA.

[17] Ioannidis M., Cerundolo V., Salio M. (2020). The Immune Modulating Properties of Mucosal-Associated Invariant T Cells. Front. Immunol..

[18] Suliman S., Murphy M., Musvosvi M., Gela A., Meermeier E.W., Geldenhuys H., Hopley C., Toefy A., Bilek N., Veldsman A. (2019). MR1-Independent Activation of Human Mucosal-Associated Invariant T Cells by Mycobacteria. J. Immunol..

[19] Jesteadt E., Zhang I., Yu H., Meierovics A., Chua Yankelevich W.J., Cowley S. (2018). Interleukin-18 Is Critical for Mucosa-Associated Invariant T Cell Gamma Interferon Responses to Francisella Species In Vitro but Not In Vivo. Infect. Immun..

[20] Arend W.P., Palmer G., Gabay C. (2008). IL-1, IL-18, and IL-33 families of cytokines. Immunol. Rev..

[21] Yasuda K., Nakanishi K., Tsutsui H. (2019). Interleukin-18 in Health and Disease. Int. J. Mol. Sci..

[22] Dinarello C.A. (2019). The IL-1 family of cytokines and receptors in rheumatic diseases. Nat. Rev. Rheumatol..

[23] Komai-Koma M., Xu D., Li Y., McKenzie A.N., McInnes I.B., Liew F.Y. (2007). IL-33 is a chemoattractant for human Th2 cells. Eur. J. Immunol..

[24] Lezmi G., Abou-Taam R., Garcelon N., Dietrich C., Machavoine F., Delacourt C., Adel-Patient K., Leite-de-Moraes M. (2019). Evidence for a MAIT-17-high phenotype in children with severe asthma. J. Allergy Clin. Immunol..

[25] Lu B., Liu M., Wang J., Fan H., Yang D., Zhang L., Gu X., Nie J., Chen Z., Corbett A.J. (2020). IL-17 production by tissue-resident MAIT cells is locally induced in children with pneumonia. Mucosal Immunol..

[26] Luo P., Deng S., Ye H., Yu X., Deng Q., Zhang Y., Jiang L., Li J., Yu Y., Han W. (2020). The IL-33/ST2 pathway suppresses murine colon cancer growth and metastasis by upregulating CD40 L signaling. Biomed. Pharmacother..

[27] Lugo-Villarino G., Maldonado-Lopez R., Possemato R., Penaranda C., Glimcher L.H. (2003). T-bet is required for optimal production of IFN-gamma and antigen-specific T cell activation by dendritic cells. Proc. Natl. Acad. Sci. USA.

[28] Le Bourhis L., Martin E., Peguillet I., Guihot A., Froux N., Core M., Levy E., Dusseaux M., Meyssonnier V., Premel V. (2010). Antimicrobial activity of mucosal-associated invariant T cells. Nat. Immunol..

[29] Ghazarian L., Caillat-Zucman S., Houdouin V. (2017). Mucosal-Associated Invariant T Cell Interactions with Commensal and Pathogenic Bacteria: Potential Role in Antimicrobial Immunity in the Child. Front. Immunol..

[30] Trottein F., Paget C. (2018). Natural Killer T Cells and Mucosal-Associated Invariant T Cells in Lung Infections. Front. Immunol..

[31] Ussher J.E., Willberg C.B., Klenerman P. (2018). MAIT cells and viruses. Immunol. Cell Biol..

[32] van Wilgenburg B., Scherwitzl I., Hutchinson E.C., Leng T., Kurioka A., Kulicke C., de Lara C., Cole S., Vasanawathana S., Limpitikul W. (2016). MAIT cells are activated during human viral infections. Nat. Commun.

[33] van Wilgenburg B., Loh L., Chen Z., Pediongco T.J., Wang H., Shi M., Zhao Z., Koutsakos M., Nussing S., Sant S. (2018). MAIT cells contribute to protection against lethal influenza infection in vivo. Nat. Commun.

[34] Fajgenbaum D.C., June C.H. (2020). Cytokine Storm. N. Engl. J. Med..

[35] Zizzo G., Cohen P.L. (2020). Imperfect storm: Is interleukin-33 the Achilles heel of COVID-19?. Lancet Rheumatol..

[36] Munitz A., Edry-Botzer L., Itan M., Tur-Kaspa R., Dicker D., Marcoviciu D., Goren M.G., Mor M., Lev S., Gottesman T. (2021). Rapid seroconversion and persistent functional IgG antibodies in severe COVID-19 patients correlates with an IL-12p70 and IL-33 signature. Sci. Rep..

[37] Flament H., Rouland M., Beaudoin L., Toubal A., Bertrand L., Lebourgeois S., Rousseau C., Soulard P., Gouda Z., Cagninacci L. (2021). Outcome of SARS-CoV-2 infection is linked to MAIT cell activation and cytotoxicity. Nat. Immunol..

