# Supplementary material for: IL-33 Enhances IFNγ and TNFα Production by Human MAIT Cells: A New Pro-Th1 Effect of IL-33
Source: Int J Mol Sci. 2021 Sep 30;22(19):10602. doi: 10.3390/ijms221910602 (PMC8508606; doi:10.3390/ijms221910602)
Supplement: Supplementary file 1 [file ijms-22-10602-s001.zip › ijms-1396183-supplementary/ijms-1396183 Table S1 revised.pdf]

Table S1. Medians, quartiles (upper and lower limit) and p values (Mann Whitney) for all measured cytokines and chemokines produced by sorted MAIT cells in response to IL-33 or IL-12 or IL-33 + IL-12 stimulations.

	**IL-33 or IL-12**			**IL-33 + IL-12**			***p* value**
Gene	**Median**	**Upper Limit**	**Lower Limit**	**Median**	**Upper Limit**	**Lower Limit**	
ACE-Hs00174179_m1	0.07	0.07	0.07	1000.00	1000.00	250.07	0.067
BAX-Hs00180269_m1	14.81	18.31	12.76	13.93	14.16	11.13	0.610
BCL2-Hs00153350_m1	1.54	2.81	1.29	1.19	1.31	1.02	0.095
BCL2L1-Hs00169141_m1	1.13	1.52	0.55	1.44	2.87	0.75	0.352
***CCL3**-Hs00234142_m1*	0.94	2.22	0.38	7.81	11.05	5.56	**0.010**
CCL5-Hs00174575_m1	75.55	92.41	32.12	44.86	63.57	40.13	0.476
CCR4-Hs99999919_m1	0.79	2.58	0.62	0.97	1.94	0.64	0.696
CCR5-Hs00152917_m1	6.46	14.68	4.46	4.29	9.45	2.98	0.257
CCR7-Hs00171054_m1	0.72	1.10	0.11	0.39	0.85	0.12	0.905
CD28-Hs00174796_m1	2.07	3.29	1.67	2.44	3.04	1.76	0.914
CD3E-Hs00167894_m1	11.97	16.02	5.65	6.78	8.42	3.56	0.257
CD40-Hs00374176_m1	0.30	3.88	0.25	0.29	0.51	0.06	0.800
***CD40LG**-Hs00163934_m1*	0.60	1.02	0.35	2.29	3.02	1.98	**0.010**
CD68-Hs00154355_m1	0.30	1.17	0.20	0.21	0.41	0.09	0.393
***CSF1**-Hs00174164_m1*	0.67	0.96	0.55	1.99	2.53	1.33	**0.032**
CXCR3-Hs00171041_m1	0.17	0.40	0.13	0.08	0.84	0.04	0.357
ECE1-Hs00154837_m1	0.18	0.38	0.08	0.24	0.28	0.12	0.967
FAS-Hs00163653_m1	1.12	1.73	0.69	1.45	2.01	1.09	0.448
FASLG-Hs00181225_m1	1.89	2.72	1.27	3.11	3.37	0.62	0.714
GNLY-Hs00246266_m1	1.96	3.93	1.21	3.60	4.66	1.48	0.610
***GZMB**-Hs00188051_m1*	0.19	0.77	0.14	26.11	51.20	8.11	**0.029**
HLA-DRA-Hs00219575_m1	0.12	0.17	0.06	0.25	1.06	0.13	0.400
HMOX1-Hs00157965_m1	0.31	0.93	0.20	1.19	1.97	0.23	0.400
ICAM1-Hs00164932_m1	0.77	2.31	0.34	1.96	4.04	0.74	0.343
ICOS-Hs00359999_m1	0.24	0.41	0.12	0.37	0.39	0.05	>0.999
***IFNG**-Hs00174143_m1*	0.27	0.85	0.09	310.79	449.07	193.32	**0.029**
IKBKB-Hs00395088_m1	2.23	2.71	1.81	1.35	2.56	0.75	0.262
IL12A-Hs00168405_m1	0.10	0.18	0.02	0.02	0.03	0.01	0.500
IL15-Hs00174106_m1	0.05	0.16	0.03	0.09	0.11	0.02	0.875
***IL2RA**-Hs00166229_m1*	0.32	1.84	0.25	6.86	12.09	5.70	**0.029**
IL8-Hs00174103_m1	0.40	1.57	0.18	0.72	1.09	0.01	>0.999
***LTA**-Hs00236874_m1*	0.48	0.69	0.21	4.34	7.46	2.73	**0.029**
NFKB2-Hs00174517_m1	3.06	6.85	2.56	9.26	12.26	6.07	0.067
PRF1-Hs00169473_m1	7.67	10.10	6.61	7.06	14.57	2.54	0.914
PTPRC-Hs00365634_g1	29.26	37.99	24.50	20.23	30.61	17.03	0.114
SKI-Hs00161707_m1	11.41	16.62	7.09	7.16	10.69	3.54	0.171
SMAD3-Hs00232219_m1	19.63	31.88	8.98	14.18	21.86	7.56	0.476
SMAD7-Hs00178696_m1	6.52	8.05	5.67	4.14	4.66	1.45	0.067
STAT3-Hs00234174_m1	10.42	13.34	7.77	6.86	11.15	4.40	0.257
TBX21-Hs00203436_m1	5.63	7.69	4.24	8.20	9.03	3.56	0.610
TGFB1-Hs00171257_m1	32.03	56.83	19.82	29.59	38.14	10.54	0.610
***TNF**-Hs00174128_m1*	6.59	16.87	5.37	21.64	32.95	12.68	**0.047**
TNFRSF18-Hs00188346_m1	0.02	0.11	0.02	0.99	1.41	0.46	0.057
VEGF-Hs00173626_m1	0.34	2.20	0.06	1.11	2.56	0.29	0.571
