# Supplementary figures and images for: IL-33 Enhances IFNγ and TNFα Production by Human MAIT Cells: A New Pro-Th1 Effect of IL-33
Source: Int J Mol Sci. 2021 Sep 30;22(19):10602. doi: 10.3390/ijms221910602 (PMC8508606; doi:10.3390/ijms221910602)

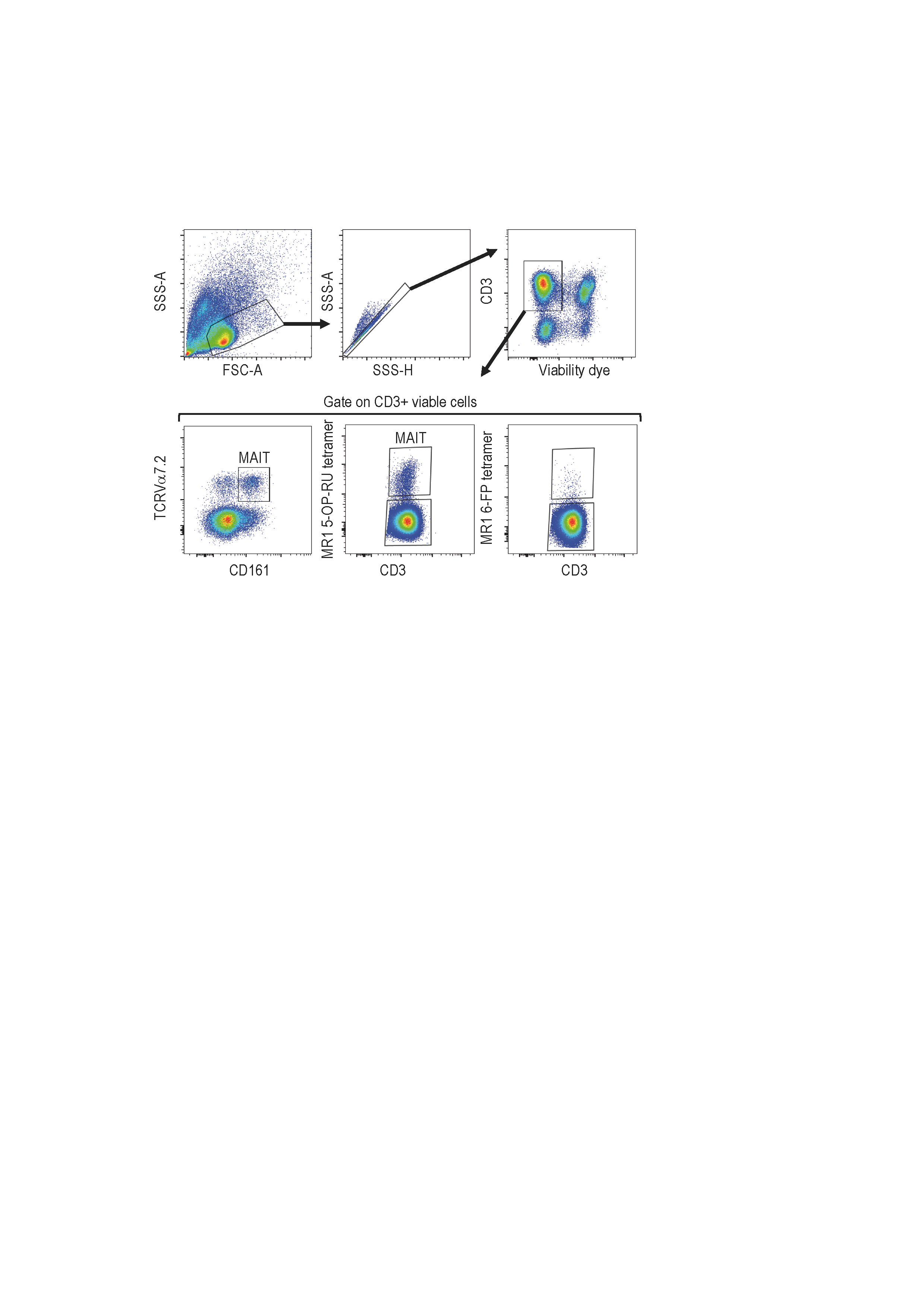

Supplement: Supplementary file 1 [file ijms-22-10602-s001.zip › ijms-1396183-supplementary/ijms-1396183 Figure S1.png]
